# Acknowledgment to Reviewers of *Journal of Imaging* in 2021

**DOI:** 10.3390/jimaging8020025

**Published:** 2022-01-26

**Authors:** Journal of Imaging Editorial Office

**Affiliations:** MDPI AG, St. Alban-Anlage 66, 4052 Basel, Switzerland

Rigorous peer-reviews are the basis of high-quality academic publishing. Thanks to the great efforts of our reviewers, *Journal of Imaging* was able to maintain its standards for the high quality of its published papers. Thanks to the contribution of our reviewers, in 2021, the median time to first decision was 21 days and the median time to publication was 47 days. The editors would like to extend their gratitude and recognition to the following reviewers for their precious time and dedication, regardless of whether the papers they reviewed were finally published:
Abdeldjalil OuahabiLaurent RisserAbraham PouliakisLee-Xieng YangAchintya BhowmikLeonardo RundoAdam MarchewkaLeopoldo Altamirano-RoblesAdam MazikowskiLirong WangAdolfo SantoroLivia Ionela BobuAhmad JalalLorenzo PutzuAkira AsanoLuc SalvoAlain TremeauLuca ArcariAldo MarzulloLuciano SilvaAleksandar KrmpotLudovic SacchelliAlessandre TengattiniŁukasz MalińskiAlessandro FantiMahmoud ElbattahAlessandro StefanoManuel CabaleiroAlessandro TonacciManuel Castejón-LimasAlex ButeanMarcelo BeckerAlex W. BarkerMarcelo FariaAlexander A. GulinMarco BottaAlexandrino GonçalvesMarco La CasciaAlexey ChubarovMarco NervoAlexey MashtakovMarco ViolaAlfred O. AnkrahMarek KojdeckiAli BakhshinejadMarek LankoszAmir BenzaouiMariana Domnica StanciuAmyar AmineMariia NazarkevychAn Cong TranMario OrtegaAna Corte-RealMariusz MaslakAnastasiia KorokhinaMariusz SzwochAndras MicsikMarko HorvatAndrea BarucciMartina Teresa BevacquaAndrea TangherloniMassimo FerriAndrei TarasovMatteo Bruno LodiAndrzej Eugeniusz StatecznyMatthias F. FroelichAndy Wai Kan YeungMehdi NassiriAnna BarnesMeng WuAnna PakulaMeropi MariAnnette FörschlerMichael MajurskiAntigoni PanagiotopoulouMichal BolaAnton TremsinMichal KawulokAntonio SarnoMichele CalìAparna BharatiMiguel MelgarejoArka DattaMihalj BakatorAsad MalikMikael BrudforsAtsushi ImiyaMikel Sesma-SaraAttila OláhMiklós BakAugusto Garcia-AgundezMilan TomaAyman Abdel-HamidMilena GeorgievaBalaji PanchapakesanMiloslav MachacekBarbara SiemiatkowskaMilvia RossiniBarbara ZitovaMing Tzer LinBekir KabasakalMirela PopaBernadette DorizziMirjeta PashaBharath NagarajaMittal MohitBirgitta Dresp-LangleyMocanu IrinaBogdan RuszczakMohamed DahmaneBo-Lin JianMohamed Sameer AbdallahBrecken BlackburnMonika MuszkietaBryan ScotneyMoreno ZanardoBurkhard SchillingerMoustafa HusseinByron Leite Dantas BezerraMuhammad AbirByunghwan JeonMuhammad Ashfaq JiskaniByung-Kuk SeoMuhammad AttamimiCarlos Cano-EspinosaMuhammad Umar ChaudhryCarmelo MilitelloMuhib Ur RahmanCatalin StoeanNageswara R. MadamanchiCatherine DisneyNaoto YagiCecile MeierNicholas PolydoridesChandrama SarkerNikolaos DikaiosChao-Ching HoNir SochenChih-Peng FanNitish KatochChris HallNuno MatelaChristian KaunertOh-Jin KwonChristof KaubaOlena IvashchenkoChuan QinOlivier LossonConstantine KotropoulosOlivier ProuxCorina NafornitaOmneya AttallahCristian StanciuOriel FrigoDalei HaoOtoniel López GranadoDaniela OriggiPaavo A. PenttiläDaniele La ForgiaPanagiotis F. LiaparinosDanut Ovidiu PopPaolo CardarelliDavid Martín GómezPascal PeterDavid McMeekinPatrick A. HelmDavid R. GreenPatrick CalletDavid S. SimonPatrick JackmanDavide SgalabernaPaulo LopesDeisy ChavesPavel RajmicDerya BirantPedro D. Frazão CorreiaDiego ValsesiaPedro Miguens MatutinoDimitris TsiktsirisPer NordbladDmitri AlekseevskyPeter BurgholzerDominik StögerPeter KazanzidesDong-Geol ChoiPhuong T. NguyenDragos SerbanPierre AmbsEberhard LehmannPierre-Luc St-CharlesEdson Eyji SanoPiotr LechEduardo Cabal-YepezPiotr S. SzczepaniakEduardo Quevedo-GutiérrezPo-Yu KaoEdward JonesPrasanna KolarEleni D. FilippakiPriyadarshini A. PattanaikElisabetta CanettaRahul UpnejaEmmanuel DufourqRami ZewailEnrico CassanoRamón QuizaEnrico Le DonneRanjit ShresthaErnest U. EkpoRaphael Dueire LinsEryk CzerwińskiRaquel ConceiçãoEvgeny BelyaevRazvan BobocFahad E. SalamhRenato CuocoloFederico GrilliniRiesz FerencFei GaoRihana PeimanFlavio BertiniRikke GadeFrancesco MarraRita Gouveia NunesFrancesco RiggiRizwan Ali NaqviFrancesco StellatoRobail YasrabFrancisco Gomez-DonosoRobert FrischholzFrank PillekampRodica HolonecG. M. Atiqur RahamanRoman ShultsGabor FichtingerRoumen KounchevGabriel Ramos LlordenRunze LiuGabriele SalvatoRute SantosGabriele VigneRyo ItabashiGanbayar BatchuluunŞaban ÖztürkGazanfar RahmathullaSabine KlingGennaro VessioSamuel Morillas GomezGerard De HaanSara Gonizzi BarsantiGermán GonzálezSara MocciaGermana LandiSebastian BudzanGhazal RouhafzaySelma RizvićGil-Jin JangSerestina ViririGiulia FestaSeung-ho LeeGiuseppe GalloSeungryul BaekGoo-Rak KwonShamim Hasnat RiponGuanqiu QiShanshan TanGuillaume HaiatShaofeng WangHaipeng LiuSharifa AlghowinemHamdi ZurqaniShashank SrivastavaHannes MareenSheryl BrahnamHelder SebastiãoShih-Chun LeeHirohiko ShimizuShusei KanieHirokazu MadokoroShyhtsong LinHomayoun NajjaranSilvia ZappavignaHongfeng YuSimona Di MeoHsuan-Ming FengSimona MoldovanuHui FangSimone TassaniHyuncheol KiSinsia A. GaoHyungchul YoonSlavenka PetrakHyung-Jeong YangSonam KaulHyunho KangStanisław HożyńHyunseok SeoStefan GutheHyunsoo LeeStefan ScheibIgor DjurovićStephan BodenIjaz Ul HaqStephen H. AndersonInes ChannoufiStuart PerryIsabella GagliardiSuah KimIvan MagdalenićSubhadip MukherjeeJacopo TessadoriSung Ha KangJames DoutchSuresh MeruguJan EggerSusana CoentroJasmina LukinacSven VogelJason BrazileSylvie ChambonJason G. ZeibelTakeharu NagaiJean SequeiraTakeshi FujiwaraJean-Jacques PoncianoTania PouliJehan-Antoine VayssadeTanmay VerlekarJennifer R. SchroederTao-Hsing ChenJhilik BhattacharyaTatiana BubbaJi Hwan ParkTeng Grace ZhangJia UddinTetsushi IkedaJian WangThanh Hai NguyenJiankang DengThorsten DerlinJimmy Aurelio Rosales-HuamaníTianfei ZhouJing QinTien-Ying KuoJinglun FengTing-To YuJoan BordoyTom BultreysJoel R. BockTomáš SkopalJohn K. DelaneyTomas ZikmundJonathan PerrinTomasz KryjakJonghoek KimTomasz LitwinJong-Uk HouTomislav KeserJorge Luis Zambrano-MartinezTomislav MaticJorge Maestre VidalTu-Liang LinJosé Alonso Solís-LemusTytus BernasJosé María Martínez-OtzetaValentin DebarnotJuan A. PeñaValentina FranceschiJuan Luis NievesVibhuti GuptaJui-yu WuVictor Fernandez NascimentoJung Sun YooVictor LeivaJunrui LiVíctor M. CampelloJyh Miin LinVincenzo ContiKai ZengVoegeli WolfgangKailun YangWassim SwailehKaisa LiimatainenWeibo LiuKalyan R. S. PerumallaWilliam McllhaggaKamlesh MistryWonsuk ChaKang-Chun PengWouter Van VerreKarim HammoudiWu ZhouKatarzyna Beata CywkaWulf GrählertKenichi OikawaXenophon ZabulisKenji ShimazoeXu GaoKhairun SaddamiYanwen SunKinjiro AmanoYassine HimeurKlaus Michael SpohrYicheng NiKogularasu SakthivelYohei InabaKosin ChamnongthaiZachary LangfordKrassimir MarkovZeno GeradtsKrzysztof GorzkiewiczZhaohui TangKrzysztof KlimaszewskiZhaohui XueLasse LensuZhengwei LiLászló SzentmiklósiZhi DengLaura AntonelliZhi QiaoLaurence Likforman-SulemZhilu LaiLaurent NajmanZiming Zhang

